# Mini-Review: Novel Therapeutic Strategies to Blunt Actions of Pneumolysin in the Lungs

**DOI:** 10.3390/toxins5071244

**Published:** 2013-07-15

**Authors:** Rudolf Lucas, Istvan Czikora, Supriya Sridhar, Evgeny Zemskov, Boris Gorshkov, Umapathy Siddaramappa, Aluya Oseghale, Jonathan Lawson, Alexander Verin, Ferenc G. Rick, Norman L. Block, Helena Pillich, Maritza Romero, Martin Leustik, Andrew V. Schally, Trinad Chakraborty

**Affiliations:** 1 Vascular Biology Center, Georgia Regents University, Augusta, GA 30912, USA; E-Mails: iczikora@gru.edu (I.C.); susridhar@gru.edu (S.S.); ezemskov@gru.edu (E.Z.); bgorshkov@gru.edu (B.G.); usiddaramappa@gru.edu (U.S.); aoseghale@gru.edu (A.O.); averin@gru.edu (A.V.); 2 Department of Pharmacology and Toxicology, Georgia Regents University, Augusta, GA 30912, USA; E-Mail: mromerolucas@gru.edu; 3 Division of Pulmonary Medicine, Georgia Regents University, Augusta, GA 30912, USA; E-Mail: jlawson@gru.edu; 4 Endocrine, Polypeptide and Cancer Institute, Veterans Affairs Medical Center and University of Miami, Miller School of Medicine, Miami, FL 33136, USA; E-Mails: ferencrick@gmail.com (F.G.R.); nblock@med.miami.edu (N.L.B.); aschally@va.gov (A.V.S.); 5 Institute of Medical Microbiology, Justus-Liebig University Giessen, D-35392, Germany; E-Mails: helena.pillich@mikrobio.med.uni-giessen.de (H.P.); martin.leustik@mikrobio.med.uni-giessen.de (M.L.)

**Keywords:** pneumolysin, permeability edema, TNF, Growth Hormone-Releasing Hormone

## Abstract

Severe pneumonia is the main single cause of death worldwide in children under five years of age. The main etiological agent of pneumonia is the G^+^ bacterium *Streptococcus pneumoniae*, which accounts for up to 45% of all cases. Intriguingly, patients can still die days after commencing antibiotic treatment due to the development of permeability edema, although the pathogen was successfully cleared from their lungs. This condition is characterized by a dramatically impaired alveolar epithelial-capillary barrier function and a dysfunction of the sodium transporters required for edema reabsorption, including the apically expressed epithelial sodium channel (ENaC) and the basolaterally expressed sodium potassium pump (Na^+^-K^+^-ATPase). The main agent inducing this edema formation is the virulence factor pneumolysin, a cholesterol-binding pore-forming toxin, released in the alveolar compartment of the lungs when pneumococci are being lysed by antibiotic treatment or upon autolysis. Sub-lytic concentrations of pneumolysin can cause endothelial barrier dysfunction and can impair ENaC-mediated sodium uptake in type II alveolar epithelial cells. These events significantly contribute to the formation of permeability edema, for which currently no standard therapy is available. This review focuses on discussing some recent developments in the search for the novel therapeutic agents able to improve lung function despite the presence of pore-forming toxins. Such treatments could reduce the potentially lethal complications occurring after antibiotic treatment of patients with severe pneumonia.

## 1. Severe Pneumonia: A Huge Medical Problem

Pneumonia kills more children, worldwide, than any other single cause of disease [1]. In addition, the elderly are highly susceptible to pneumonia. In the United States, more than five million cases of community-acquired pneumonia (CAP) are diagnosed annually, with 7% of them being fatal [2]. *Streptococcus pneumoniae* (*Pneumococcus*) represents the main etiological agent of severe pneumonia and accounts for up to 45% of all cases. In spite of antibiotic treatments, *Pneumococcus* vaccines, and supportive health care, many patients still die from complications [1,2], the most important one of which is pulmonary permeability edema. Intriguingly, this complication can occur days after the initiation of antibiotic therapy, when tissues are pathogen-free and the pneumonic process is clearing. This edema formation correlates with the presence of the bacterial virulence factor, pneumolysin (PLY) [3].

## 2. Importance of Pulmonary Barrier Integrity

The lung represents a crucial interface between the external atmosphere and the energy-requiring cells of our body. As such, it must contain a selective barrier to the movement of O_2_, crucial for mitochondrial electron transport-mediated ATP generation, and of the waste product CO_2_ between the outside environment and the blood. This barrier is composed of three components: the basement membrane, the alveolar epithelial cells at the air interface, and the capillary endothelial cells at the blood interface, all of which are critical to the normal pulmonary function. Both, alveolar epithelial and capillary endothelial barriers, are regulated by a tight balance between centripetal and centrifugal intracellular forces. These are provided by contractile mechanisms and the elements opposing contraction, respectively. The elements opposing barrier dysfunction include tethering complexes, involved in cell-cell and cell-matrix contacts, as well as systems granting cell rigidity and preventing cell collapse, such as actin filaments, microtubules, and intermediate filaments [4]. Binding to cholesterol by pneumolysin (PLY), the main virulence factor of *S. pneumonia*, or by the homologous cytolysin listeriolysin (LLO) (from *Listeria monocytogenes*), is followed by oligomerization and membrane pore formation, resulting in a rapid increase in intracellular Ca^2+^ and diacylglycerol levels [5], and in severe pulmonary hyperpermeability [6,7].

A rise in cytosolic Ca^2+^, as caused by both PLY and LLO, has been proposed to be the initial pivotal signal preceding endothelial cell contraction, since it can activate key signaling pathways that mediate cytoskeletal reorganization (through myosin light chain (MLC)-dependent contraction) and disassembly of VE-cadherin at the adherens junctions [8]. Rho (Ras homologous) GTP-binding proteins, which comprise multiple members of the Rho, Rac, and Cdc42 subfamilies, are involved in the regulation of a variety of cellular processes [9]. RhoA and Rho-associated kinase may directly catalyze myosin light chain (MLC) phosphorylation, or act indirectly via inactivation of MLC phosphatase [10,11,12], to induce cell contraction and endothelial barrier disruption. In turn, endothelial barrier enhancement is associated with Rac1-mediated formation of F-actin, increased association of focal adhesion proteins, and enlargement of intercellular adherens junctions [10]. Thus, a precise balance between RhoA- and Rac1-mediated signaling is essential for endothelial barrier regulation. 

The Ca^2+^-dependent protein kinase C (PKC) isoform, PKC-α, was suggested to play a critical role in initiating endothelial cell contraction and disassembly of VE-cadherin junctions [13,14,15].

The NADPH oxidases Nox2 and Nox4 are major sources of reactive oxygen species (ROS) in endothelial cells and are implicated in redox-sensitive signaling pathways that influence endothelial cytoskeletal organization and permeability [16].

Apart from inducing RhoA activation [15], PKC-α activation was also recently shown to upregulate Nox 4 mRNA expression in human endothelial cells [17].

Maintenance of the endothelial barrier also requires a basal level of nitric oxide (NO), regulated by endothelial NOS (eNOS) [18]. Both, the lack of NO and high NO levels destabilize interendothelial junctions [18,19,20].

## 3. Pneumolysin: Structure and Activities

PLY is found in all clinically relevant isolates of *S. pneumoniae* and is classified as one of its most important virulence factors [21,22]. Although isolates of serotypes that caused outbreaks of pneumococcal disease have been found to carry non-hemolytic PLY variants, there is no direct clinical evidence that *S. pneumoniae* without a functional toxin is pathogenic [23]. As of its discovery as a hemolytic factor [24], several characteristics typical for the cholesterol-dependent cytolysin (CDC) family, as cholesterol dependence and increased activity under reducing conditions [25,26,27,28], have been identified. CDCs are exclusively produced by Gram-positive bacteria and of all 25 known CDCs, generated by 27 bacterial species, PLY is the only one that is not secreted into the extracellular medium (reviewed in [29]). PLY has been cloned, sequenced, and purified [30,31,32], in order to better understand its role in pneumococcal pathogenesis. 

PLY is a 53 kDa cytoplasmic thiol-activated toxin with cytolytic and complement-activating properties. CDCs, including PLY, at high concentrations non-specifically lyse eukaryotic cells. The common features of all CDCs include their dependence on cholesterol in membranes, as they are otherwise unable to bind and perforate them. Another common feature of CDCs is their ability to form very large pores, which can consist of up to 50 toxin monomers, with maximum diameters of up to 45 nm. 

The various CDCs have significant levels of amino acid homology and share the same mode of action. The common structure of CDCs is an elongated, β-sheet-rich shape that can be separated into four domains, the fourth one of which has been recognized to be responsible for the membrane binding. Within this fourth domain, all CDCs share a highly conserved motif (ECTGLAWEWWR), the so-called undecapeptide, which is necessary for recognition and binding to cholesterol in the membranes [3,7,33,34,35].

There has recently been progress in the understanding of how PLY binds to cholesterol-containing membranes. It was reported that domain four of PLY interacts directly with the 3β-hydroxyl group at the C-3 atom of cholesterol, which could explain the constitutive dependency of PLY on cholesterol in the membranes that are to be perforated [36]. Through binding experiments with truncated toxin subunits and different carbohydrates, it was also revealed that domain four has a high affinity for mannose, suggesting that mannose-containing glycoproteins or glycolipids might be receptors for the recognition of target cells.

Monoclonal antibodies that are able to neutralize the cytolytic activity of CDCs were found to inhibit only pore formation, but not membrane binding and oligomerization into a ring-shaped prepore [37,38]. This indicates a stepwise process that can be stopped after membrane binding and oligomerization of toxin monomers, but before pore formation. 

## 4. PLY Interactions with the Immune System

Interestingly, PLY is not actively secreted, but stored inside the bacterial cell. It is released during bacterial lysis, as from antibiotic treatment [39,40], or by the action of the pneumococcal virulence factor autolysin A (lytA) [41,42,43,44]. The activation of lytA can be triggered by the immune system, antibiotics or other bacterial virulence factors [45,46]. Moreover, it is active when the bacteria reach a plateau in their growth phase *in vitro* [44]. This seemingly suicidal behavior favors bacterial survival in the host because PLY has many immunomodulatory effects. It was found to inhibit migration, respiratory burst, degranulation, and other bactericidal activities in polymorphonuclear leukocytes and monocytes [47,48].

However, in comparison to Gram-negative bacteria-derived lipopolysaccharide (LPS), for example, PLY induces only a modest inflammatory response [49]. The toxin can nevertheless induce activation of the NLRP3 inflammasome, thereby activating immune defense mechanisms in the host [50], and also seems to be responsible for the production of type I interferons in macrophages after phagocytosis of pneumococci [51]. It was reported that PLY-mutants and non-hemolytic strains of pneumococci do not trigger this cytokine release, but the role of the toxin in these mechanisms is still unclear [51,52,53]. 

All these findings indicate a modest role for PLY in the modulation of inflammatory reactions during infection with *S. pneumoniae*. These results however also indicate that PLY’s direct effects on the alveolar-capillary barrier predominate over its effects on resident or recruited phagocytic cells [49].

## 5. PLY Compromises the Barrier Function of the Lung

PLY can be released in massive amounts during, and following, antibiotic treatment of pneumococcal pneumonia patients [39,40]. Instillation of purified PLY into rat lungs causes reactions very similar to those of pneumonia, such as neutrophilic alveolitis and impaired pulmonary barrier function [54]. A different study found that intranasal application of recombinant toxin in mice lead to significant lung microvascular leakage but lacked the expected neutrophil lung infiltration within the first 12 h [7]. This indicates that the tissue damage and loss of function was independent from neutrophil influx at early stages, which is supported by a previous study [49]. 

Recently, it was discovered that platelet-activating factor (PAF) and associated downstream signaling pathways play a role in the PLY-induced development of acute lung injury [55]. Toxin treatment in mouse lungs caused a dose-dependent increase in pulmonary vascular resistance and increased PAF production. Downstream of PAF, phosphorylation of myosin light chain (MLC), triggered by increased levels of thromboxane B2, phosphatidylcholine-specific phospholipase C, and protein kinase C (PKC) activation, induced pulmonary vasoconstriction. In other mouse experiments, PLY-deficient *S. pneumoniae* mutants are cleared faster from the respiratory tract and have a significantly reduced lethality following intranasal application, as compared to toxin-producing strains [53,55]. 

While higher amounts of PLY instantly lyse and kill cells in an unspecific manner, sub-lytic concentrations can interfere with cell signaling events by inducing an influx of extracellular Ca^2+^ and disturbances of osmotic homeostasis through toxin-induced pores. This can activate barrier-disruptive host enzymes, such as rho-associated kinase (ROCK), and Ca^2+^-dependent PKC isozymes, such as PKC-α [13,14,15]. Blunting the PLY-induced Ca^2+^-influx, as by use of lanthanum chloride, was shown to significantly blunt the effects of sub-lytic PLY concentrations on endothelial barrier dysfunction [56]. Following Ca^2+^-influx-mediated activation, these enzymes can activate barrier-disruptive pathways, including myosin light chain (MLC)-dependent mechanisms and microtubule de-polymerization. The latter can cause disassembly of adherens junction proteins, such as VE-cadherin in endothelial cells [14]. 

A recent study reported that PLY binds cellular actin with high affinity, an activity that is dependent on the pore forming capacity of the toxin [57]. The same research group also showed that the toxin is internalized after binding to the cell membrane, indicating a repair mechanism that allows cells to withstand and recover from toxin concentrations that are not immediately lytic [57]. 

Aside from inducing MLC phosphorylation and microtubule disassembly, PLY also increases the activity of arginase 1, an enzyme competing with endothelial nitric oxide synthase (eNOS) for the common substrate L-arginine. Arginase 1 activation can, as such, cause endothelial nitric oxide (eNOS) dysfunction [56] and reduced NO generation in human lung microvascular endothelial cells (HL-MVEC) [58]. The latter activity also correlates with increased endothelial permeability, as the arginase inhibitor BEC is able to partially blunt PLY-induced hyperpermeability *in vitro.* Moreover, mice with reduced arginase 1 expression have a significantly reduced sensitivity towards PLY-induced capillary leak [58]. Thus, inhibition of MLC and VE-cadherin phosphorylation, or arginase activation, represent important potential targets for therapeutic candidates intended to diminish PLY-induced capillary leak. 

Apart from negatively affecting capillary endothelial barrier function, PLY also alters alveolar permeability and affects epithelial tight junction integrity [59]. At high concentrations, the toxin is highly cytotoxic to alveolar epithelial cells, causing cytoplasmic blebbing, mitochondrial swelling, and cell death [60]. Its hemolytic activity also impairs ciliary function in airway epithelial cells [61]. Moreover, a significant increase in the tight junction protein claudin 4 expression during acute lung injury was detected in type II alveolar epithelial cells and may represent an adaptive response to limit airspace edema formation and allow higher rates of alveolar liquid clearance, as claudin 4 decreases paracellular permeability to large molecules [62]. 

Our preliminary data have shown that PLY not only impairs pulmonary barrier function, but also blunts amiloride-sensitive Na^+^ uptake, crucial for alveolar liquid clearance, in H441 cells [63]. The capacity of the lungs to clear liquid was demonstrated to inversely correlate with mortality and morbidity in patients suffering from acute lung injury and acute respiratory distress syndrome [64]. Taken together, PLY-induced pulmonary dysfunction is characterized by both impaired capillary-alveolar epithelial barrier integrity and dysfunctional activity of the epithelial sodium channel (ENaC) in the epithelial barrier. 

As no standard treatment currently exists to counteract PLY-mediated acute lung injury, the search for novel protective pathways, which can interfere with most of these crucial mediators of edema is, therefore, of high clinical importance. In the next sections we will therefore discuss some recent developments in this field.

## 6. Does the Brain Protect Our Lungs During Pneumonia? The Unexpected Story of Growth Hormone-Releasing Hormone

The main function of Growth Hormone-Releasing Hormone (GHRH), generated in the hypothalamus [65,66], appears to be the stimulation of Growth Hormone production by the pituitary, which expresses full-length GHRH receptors. Intriguingly, mRNA for the ligand GHRH, and its receptor splice variants SV-1 (bioactive) and SV-3 (scavenger), were recently found to also be expressed in peripheral tissues, such as in the lung capillaries [63,67]. The sequence of the 29 *N*-terminal amino acid residues of GHRH possesses full biological activity and, thus, constitutes the core functional peptide moiety for the development of agonists of GHRH. JI-34, JI-36, and JI-38 are designations of synthetic analogs of this core peptide chain, which are up to 60 times more potent than native GHRH [68].

When GHRH or GHRH receptor agonists bind to their receptor, this causes the activation of the closely associated heterotrimeric Gα_S_-protein. Upon this activation, the dissociated α subunit of Gα_S_ directly stimulates adenylate cyclase, leading to increased cAMP generation in the membrane compartment, which in turn activates the barrier-protective protein kinase A (PKA) [69]. It should be noted here that adenylate cyclase also exists in a soluble form (sAC10) in the cytosol of HL-MVEC, leading to a Ca^2+^ and bicarbonate-sensitive cAMP pool, regulated by phosphodiesterase 4, which paradoxically appears to increase endothelial permeability [70]. 

We have shown that the GHRH-R synthetic agonist, JI-34, protects lung microvascular endothelial cells, which express mRNA for GHRH, as well as for the GHRH-R splice variants SV-1 and SV-3, from the hyperpermeability induced by the pneumococcal virulence factor, PLY [63]. JI-34 exerts this effect, at least partially, by means of reducing PLY-mediated MLC and VE-cadherin phosphorylations, two events contributing to the endothelial barrier dysfunction. 

The Gα_S_-mediated activation of adenylate cyclase, leading to cAMP generation and PKA activation, is crucial for the protective effect of the GHRH agonist [63]. Increased generation of cAMP has been demonstrated to attenuate both epithelial and endothelial barrier dysfunction [71,72,73]. In endothelial cells, cAMP potentiates VE-cadherin-mediated cell-cell contacts, causing enhanced endothelial barrier function [72]. Consequently, the inhibitory activity of the GHRH agonist, JI-34, on PLY-mediated VE-cadherin loss [63] can at least be partially explained by its capacity to increase cAMP generation, upon binding to the SV-1 receptor, expressed in HL-MVEC. In addition, it was demonstrated that PKA activation in the endothelium can result in inhibition of Rho-dependent kinase (ROCK), by direct phosphorylation of Rho GDP-dissociation inhibitor (RhoGDI) and prevention of the release of RhoA from the RhoA-RhoGDI complex [73].

Our recently obtained data also indicate that the GHRH agonist, JI-34, in addition to strengthening barrier integrity in PLY-treated endothelial monolayers, is also able to restore PLY-induced Na^+^ uptake impairment, crucial for alveolar liquid clearance, in a cAMP-dependent manner [63]. Cyclic AMP-dependent stimulation of Na^+^ influx across H441 or type II alveolar epithelial cell confluent monolayers was shown to result from activation of an amiloride-sensitive apical Na^+^ conductance [74,75]. In this context, it is interesting to note that cAMP-mediated activation of PKA leads to increased expression of the crucial α-subunit of the epithelial sodium channel ENaC [76], which is the main regulator of apical Na^+^ uptake in type II alveolar epithelial cells. 

It is plausible that the generation of cAMP, which occurs as a direct effect of ligand binding to the SV-1 receptor, potentiates PKA signaling in both epithelial and endothelial cells. The latter can then counteract and override the potentially negative PKC-mediated effects [77]. In sharp contrast to the barrier-stabilizing effects observed with the GHRH agonist JI-34, the GHRH antagonist, MIA 602 [78], reduces monolayer resistance in HL-MVEC, which expresses mRNA for the bioactive splice variant 1 (SV-1) of the GHRH-R [63]. GHRH-R signaling might thus be required for maintenance of basal microvascular endothelial barrier integrity, which requires further investigation. 

**Figure 1:**
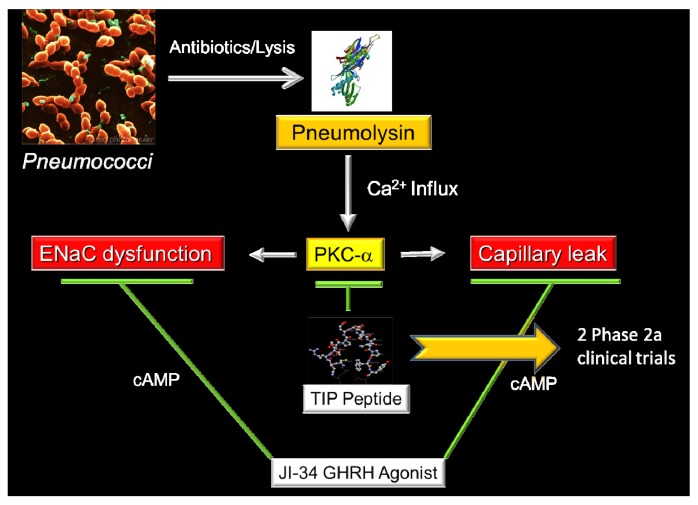
Antibiotic-induced release of pneumolysin (PLY) by *S. pneumoniae* causes a rapid influx of Ca^2+^, which activates Protein Kinase C-α. This enzyme is involved in the induction of hyperpermeability in the capillary endothelium and, moreover, causes a reduced expression and activity of the epithelial sodium channel (ENaC) in type II alveolar epithelial cells. Our preliminary data have shown that two peptides derived from the body’s own mediators, *i.e.*, the TNF-derived TIP peptide (chapter 7) and the Growth Hormone-Releasing Hormone-derived agonist JI-34 (chapter 6) can restore barrier integrity and ENaC function in the presence of PLY, in a cAMP-independent and -dependent manner, respectively.

In summary, as indicated in Figure 1, agonists of GHRH, in a cAMP-dependent manner, can counteract negative effects of PLY on both ENaC function and capillary resistance. Elucidation of the mechanism by which GHRH-R signaling regulates pulmonary endothelial barrier function can proceed to the concept that hypothalamic hormones play a role in regulating lung function during pneumonia. Moreover, since the synthetic GHRH agonists have a longer serum half-life than the hypothalamic GHRH hormone itself [68], they can deliver a more potent signal for damaged lungs and provide the basis for novel treatment options for pneumonia and acute lung injury.

## 7. The Lectin-like Domain of Tumor Necrosis Factor: A Promising Therapeutic Candidate for the Treatment of Permeability Edema

Although it is generally assumed that cytokines solely exert their activities upon activating their respective receptors, this does not seem to be completely true in the case of TNF. Indeed, in contrast to lymphotoxin-α, which has a highly homologous three-dimensional structure and can bind to both TNF receptors, TNF was shown to exert a lytic activity in purified long slender blood stream forms of African trypanosomes [79]. Interestingly, the trypanolytic effect of TNF on purified *T. brucei brucei* AnTat 1.1 parasites cannot be inhibited by complexing the cytokine with the soluble TNF receptor 1, which blocks all TNF receptor-mediated effects [79,80]. 

TNF exerts a lectin-like activity, permitting its binding to glycoproteins such as uromodulin (KD = 10^−10^ M), a glycoform of Tamm-Horsfall protein, found in the loops of Henle, of pregnant women. Interestingly, uromodulin was shown to be able to bind the pro-inflammatory cytokines, IL-1β, IL-2, and TNF, proposed as a mechanism to clear excessive levels of these cytokines from the circulation during pregnancy [81]. Since uromodulin-bound TNF was still able to exert cytotoxic effects in L929 fibrosarcoma cells, it was proposed that the lectin-like domain of TNF has to be spatially distinct from its receptor binding sites [81]. 

Our observations that (i) specific oligosaccharides, such as *N*,*N*'-diacetylchitobiose, as well as branched trimannoses, which are known to bind to the lectin-like domain of TNF, are able to inhibit the trypanolytic, but not the cytotoxic activity of TNF in L929 cells, and that (ii) lectins with a similar oligosaccharide specificity as TNF, such as Urtica Dioica Agglutinin, but not those with a different specificity, block the trypanolytic effect of TNF [79], lead to the hypothesis that the lectin-like domain of TNF is responsible for its trypanolytic activity. This was confirmed by the observation that TNF is able to bind to the Variant Surface Glycoprotein of the trypanosomes, upon which the cytokine is endocytosed and transported to the lysosomes, where intra-lysosomal rupture finally causes the trypanolysis to occur [82]. 

Molecular graphics comparisons of tertiary structures of TNF (trypanolytic), and the highly homologous lymphotoxin-α (non-trypanolytic), lead us to propose a dissimilar structure that could be responsible for the lectin-like activity. This structure, which is present at the tip of the TNF molecule, could be mimicked by a circular 17 amino acid peptide, which we called the TIP peptide. Antibodies to this peptide were able to inhibit the trypanolytic activity and moreover, the TIP peptide itself was shown to exert trypanolytic activity [79]. Three amino acids, *i.e.*, one threonine and two glutamic acids, were shown to be crucial for this activity. 

Apart from inducing trypanolysis, the lectin-like domain of TNF, mimicked by the TIP peptide, was also shown to activate sodium transport in alveolar epithelial cells, and microvascular endothelial cells [83,84]. This is not a direct effect of TNF, but, rather, involves the TNF-mediated activation of endogenous amiloride-sensitive sodium channels. As the uptake of sodium in type II alveolar epithelial cells is a crucial event in alveolar liquid clearance, an event necessary to assure proper gas exchange in the alveoli, we and others have evaluated the capacity of the lectin-like domain of TNF, mimicked by the TIP peptide, to activate edema reabsorption in *in situ*, *ex vivo*, and *in vivo* flooded rat, mouse, and rabbit lung models. As such, it was found that the peptide is able to efficiently activate edema reabsorption, to the same extent as the β2 adrenergic agonists, for example, in the absence of any pro-inflammatory activity [85,86]. Interestingly, the lectin-like domain of TNF probably performs this activity in a catecholamine-independent manner [83]. Moreover, whereas wt hTNF rather decreased lung liquid clearance (LLC) in a rat hydrostatic edema model *in vivo*, probably because of the TNF receptor 1-mediated effects on the expression and function of the epithelial sodium channel [87], complexing the cytokine with its soluble TNF receptor 1 shifted this activity towards an activation of LLC, and the oligosaccharide *N*,*N*'-diacetylchitobiose, to which the lectin-like domain of TNF binds, can reduce this activity to basal levels [86]. 

These data suggest that, physiologically, the activity of the lectin-like domain of TNF represent a function of the cytokine that is hidden by the adverse effects mediated by the TNF receptor binding sites [88]. However, in the presence of the soluble TNF receptors, this lectin-like activity can prevail, thus, providing an explanation of why positive effects of TNF in alveolar liquid clearance were mainly reported in infection models, which are characterized not only by increased levels of TNF, but also of its soluble receptors [89]. 

**Figure 2:**
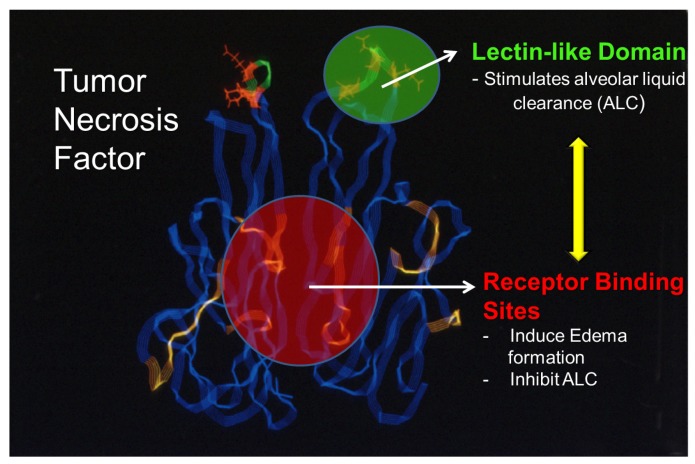
TNF: a “moonlighting” cytokine. Whereas the TNF receptor 1 binding sites within the TNF homotrimer mediate edema formation and blunt edema reabsorption, the lectin-like domain of the same cytokine, rather, activates ENaC function and as such promotes alveolar liquid clearance.

Taken together, these results indicate that in murine models of hydrostatic edema, the receptor binding sites of TNF inhibit, whereas its lectin-like domain activates edema reabsorption [86,88]. As such, as depicted in Figure 2, there are at least two spatially distinct functional domains in TNF exhibiting opposing effects in terms of edema formation and clearance. On the one hand, there is the TNF receptor 1 binding site, which inhibits edema reabsorption or contributes to edema formation [90] and, on the other hand, there is the lectin-like domain of TNF that has a high affinity for specific sugar groups, such as *N*,*N*'-diacetylchitobiose and which activates edema reabsorption. 

In contrast to the situation in hydrostatic edema, during acute lung injury, such as in severe pneumonia, the alveolar-capillary barriers can become disrupted or leaky, leading to an infiltration of neutrophils and factors contained in the blood into the alveoli, for example. Other’s recent data have indicated that also in LPS/*S. aureus* α toxin-treated isolated perfused rabbit lungs *ex vivo* the TIP peptide significantly increases fluid reabsorption [91]. Moreover, the TIP peptide prevents ischemia-reperfusion injury upon rat left lung isotransplantation *in vivo*, by means of blunting hypoxia-reoxygenation-induced ROS generation in the transplanted lungs [92].

Our recent data have demonstrated that the TIP peptide is capable of blunting the activation of the enzymes protein kinase C-α, which promotes permeability, and arginase 1, which competes with endothelial nitric oxide synthase, upon intratracheal pneumolysin instillation in mice *in vivo* and in human lung microvascular endothelial cell monolayers *in vitro*. As such, the peptide can prevent microtubule rearrangement and loss of VE-cadherin adherens junctions in the lung capillaries (Figure 1) [58]. 

Our research has the objective of further elucidating the mechanism of action of the TIP peptide (also termed AP301 peptide), that mimics the cytokine’s lectin-like domain and that is currently being tested in two phase 2a clinical trials, in patients with the acute respiratory distress syndrome (ARDS) and following lung transplantation, respectively. 

## 8. Conclusions

Although much work remains to be done in this field, recent research results demonstrate that it will be possible to identify and synthesize therapeutically appealing peptides derived from autologous proteins, such as cytokines and hormones, for the treatment of the pulmonary edema, associated with the effects of the pneumococcal virulence factor, pneumolysin.
